# ‘Back-and-Forth Stomach’ CT Imaging Findings of a Pathophysiologic Entity Causing Acute Gastric Volvulus

**DOI:** 10.3390/tomography8010019

**Published:** 2022-01-21

**Authors:** Antonio Jesús Láinez Ramos-Bossini, Eduardo Ruiz Carazo, María Dolores Rabadán Caravaca

**Affiliations:** 1 Department of Radiology, Virgen de las Nieves University Hospital, 18014 Granada, Spain; edu.eruiz@gmail.com; 2 Department of Radiology, Infanta Margarita Hospital, 14940 Cabra, Spain; mariadolrc@gmail.com

**Keywords:** gastric volvulus, computed tomography, back-and-forth stomach, emergency, radiology

## Abstract

Background: Gastric volvulus (GV) is a life-threatening emergency condition that prompts emergent surgical management. With the advent of high-resolution computed tomography (CT), the role of radiologists in its diagnosis has become essential. Although many cases of GV have been described in the literature, its pathophysiology is still poorly understood. In addition, there is substantial terminological confusion with associated entities such as paraesophageal hernia, upside-down stomach, organo-axial or chronic GV. Methods: We conducted a retrospective review of clinical, radiological findings and other relevant data for seven patients with previous radiological diagnoses of a large hiatus hernia who presented with acute GV to the emergency department of our institution. We report data on age, sex, medical history, clinical presentation, imaging, treatment and outcomes for each case. Results: The CT findings at acute presentation showed the antrum lying above the diaphragm and dilated fundus below the diaphragm. By comparing the position of the stomach at acute presentation with previous imaging examinations, we confirmed a hypothesis put forward by a few authors decades ago that re-herniation of the gastric fundus into the abdomen is a common pathophysiologic trigger leading to acute GV. This hypothesis has not been supported by modern imaging examinations. Conclusions: We have provided imaging evidence supporting that the pathophysiology of many GVs is based on caudal re-descent of hiatal hernia into the abdominal cavity. Given the terminological disparity used in the literature in this context, we believe it appropriate to introduce and extend the term ‘back-and-forth stomach’ to refer to this type of GV.

## 1. Introduction

Gastric volvulus (GV) is a rare complication secondary to twisting of the stomach more than 180° around its own axis, either transversally or longitudinally, resulting in a closed-loop obstruction [1,2,3,4]. The first reports of this entity date back to the end of the 19th century [5]. Since then, numerous case reports and series have been published, contributing to a better understanding of GV. Although sociodemographic data are limited [6], no significant differences in its incidence have been described in terms of sex or race, and it is more frequent in the fifth decade of life [2,7,8,9]. Because the clinical presentation of GV is nonspecific, imaging examinations are required for appropriate diagnosis. Multi-detector computerized tomography (MDCT) scanners offer excellent temporal and spatial resolution with multiplanar reformatting capability, high image quality and diagnostic reliability. Accordingly, MDCT is currently considered the ‘gold standard’ in the diagnosis of GV [10,11]. 

Several classification systems of GV have been described in the literature, emphasizing different aspects such as the degree of rotation (partial, <180° vs. total, >180°), etiology (primary vs. secondary), or time from onset of symptoms (acute vs. chronic) [12]. However, the most relevant classification of GV, which was described as early as 1912 [13] and completed in 1940 by Singleton [14,15], is topographic. This classification is based on the main axis of rotation and differentiates two main types of GV, namely organoaxial and mesenteroaxial. The former group is more frequent [16] and characterized by a longitudinal axis of rotation (imaginary line that joins the gastroesophageal and antropyloric junctions), so that the greater curvature lies above the lesser curvature. The latter group is defined by rotation around the axial axis of the stomach (imaginary line joining the greater and lesser curvatures), so that the gastroesophageal junction (GEJ) lies below the gastroduodenal junction. There is also a less frequent (incidence around 2%) form of GV in which rotation occurs in both rotational axes [16]. Although the topographic classification is useful, imaging findings are often confusing and difficult to interpret, leading to over- and under-diagnosis of GV [6]. Moreover, they provide little information regarding the underlying pathophysiological mechanism.

In this article, we describe a series of acute GV occurrences diagnosed by CT which were caused by re-herniation of previous large hiatal hernias (HHs) into the abdomen. We review patients’ characteristics, imaging findings and the previous literature on the topic, discuss terminological inconsistencies and review the pathophysiological mechanism underlying this type of GV.

## 2. Materials and Methods

An observational study designed from a retrospective case series was conducted at the Virgen de las Nieves University Hospital (Granada, Spain). We consulted our hospital’s radiological information system database and reviewed all CT exams in which the radiology report contained the terms ‘gastric’ or ‘stomach’ and ‘volvulus’. The study period was from 1 January 2010 to 31 October 2021. Of the total of eligible CT exams (43 studies), we excluded redundant examinations (i.e., performed on the same patient) and selected those with prior imaging exams demonstrating an HH. Accordingly, we report the clinical, radiological findings and other relevant data for seven patients with a previous radiological diagnosis of large HH who presented with acute GV to the emergency department of our institution. We report data on age, sex, medical history, clinical presentation, imaging findings, management and patient outcomes for each case.

CT studies were performed on 16 and 64 MDCT scanners with axial slice thickness of 1 mm. Contrast-enhanced CTs, when performed, were acquired at 60 s with contrast bolus tracking and flow rates of 4 mL/s.

## 3. Results

**Figure 1:**
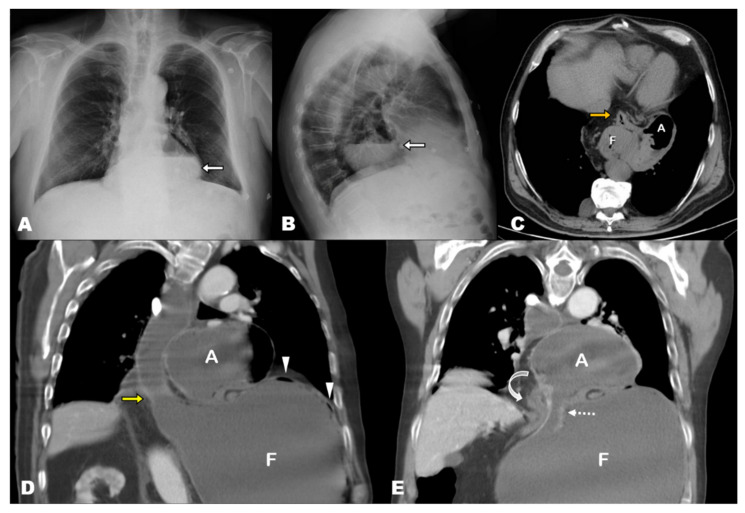
(**A**,**B**) Chest radiography 3 years prior to acute presentation, posteroanterior and lateral views. Large retrocardiac round radiolucency containing an air-fluid level, which corresponds to a sliding hiatal hernia containing the entire stomach. (**C**) Axial-oblique MPR non-contrast CT image corroborates the presence of the entire stomach and first duodenal segment (orange arrow) in the mediastinum. (**D**,**E**) Contrast-enhanced at the acute presentation, coronal-oblique MPR views. Marked dilatation of the stomach and esophagus. The antrum (A) lies above the diaphragm, and the fundus (F) is herniated through the esophageal hiatus (yellow arrow). Note the hernia neck, dotted arrow in (**E**). The antropiloric junction originates from the posterior part of the antrum (bending arrow in (**E**) and lies to the right side of the esophageal hiatus. These findings indicate that the fundus, which was previously located in the mediastinum, re-herniated into the abdominal cavity, triggering the gastric volvulus. Note the absence of nasogastric tube, which could not be inserted in this patient.

**Figure 2:**
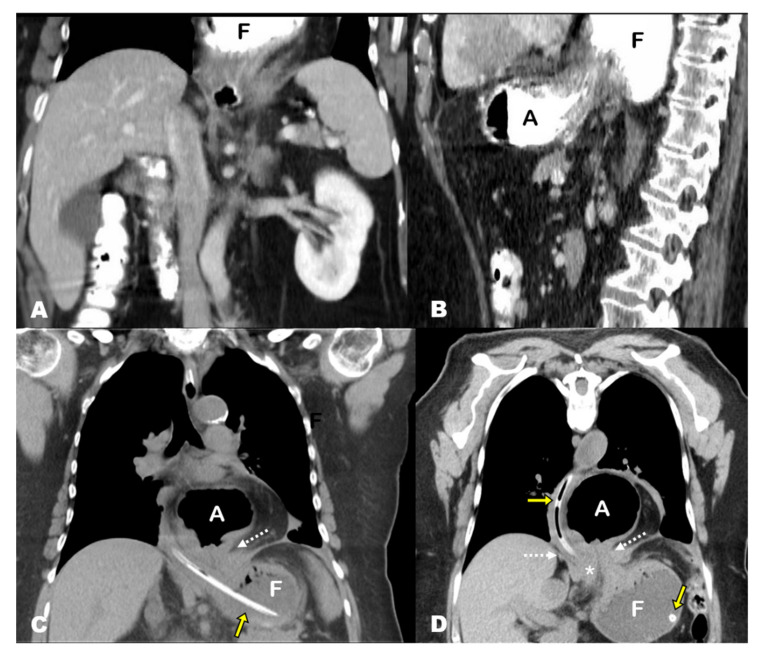
(**A**,**B**) Contrast-enhanced abdominal CT after oral contrast administration 5 years prior to the acute presentation, coronal and sagittal MPR views. The superior slices of the images show a hiatal hernia containing the gastric fundus (F). The antrum lies below the diaphragm, and there is no evidence of the esophagogastreal junction because it lies superiorly and was not included in the study (sliding hernia). (**C**,**D**) Non-contrast chest CT scan, coronal and oblique-coronal MPR views. The antrum (A) lies above the diaphragm, and the fundus (F) is re-herniated through the esophageal hiatus (note the hernia neck, dotted arrow). The antropiloric junction originates from the posterior part of the antrum and lies to the right side of the esophageal hiatus (asterisk). These findings indicate that the fundus, which was previously located in the mediastinum, re-herniated into the abdominal cavity, triggering the gastric volvulus. Note the nasogastric tube in the fundus (yellow arrows).

**Figure 3:**
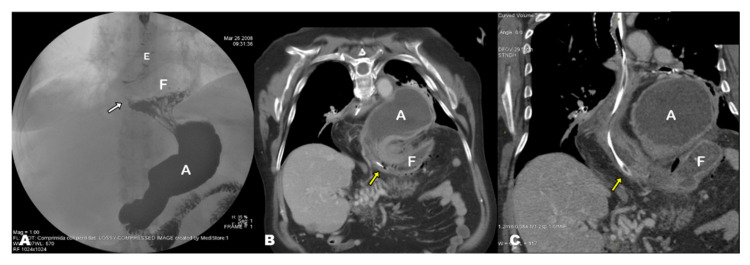
(**A**) Barium swallow performed 10 years prior to acute presentation, anteroposterior view. The gastroesophageal junction (white arrow) along with part of the gastric fundus (F) are located above the diaphragm. These are typical findings of sliding hiatal hernia. (**B**,**C**) Contrast-enhanced abdominal CT image at the acute presentation, coronal-oblique MPR view. Acute gastric volvulus showing the antrum (A) and fundus (F) above and below the diaphragm, respectively. Note the nasogastric tube (yellow arrows) at the level of the diaphragm. E, esophagus.

**Figure 4:**
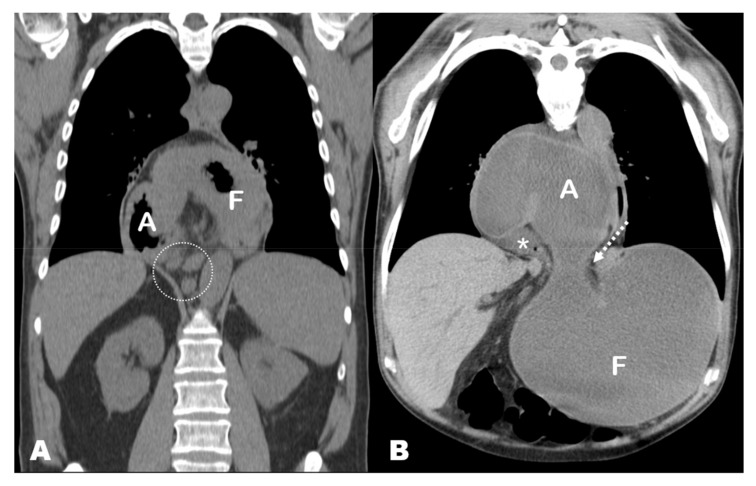
(**A**) PET-CT exam performed 1 month prior to the acute presentation, coronal MPR view. The entire stomach is located in the mediastinum and shows an abnormal position with the lesser curvature downwards, the antrum (A) in the right side and the fundus (F) in the left side. This has been referred to as ‘upside-down stomach’, but it is simply an exaggerated form of sliding hiatal hernia with horizontal rotation of the stomach. Note the presence of retrocrural lymphadenopathies (circle) related to a previously known patient’s lymphoma. (**B**) Non-contrast chest CT exam at the acute presentation, coronal oblique MPR view. The antrum lies above the diaphragm, and the fundus (F) is herniated through the esophageal hiatus (note the hernia neck, dotted arrow). The antropiloric junction originates from the posterior part of the antrum and lies to the right side of the esophageal hiatus (asterisk). These findings indicate that the fundus, which was previously located in the mediastinum, re-herniated into the abdominal cavity, triggering the gastric volvulus.

Three of the seven patients with acute GV in our series were women. The mean age of patients was 70.57 years (standard deviation [SD], 12.38; range, 47–85 years). The mean time from diagnosis of HH was 4.73 years (SD, 3.23, range, 1 month–10 years), and these patients were diagnosed with different imaging techniques. Specifically, four HHs were diagnosed with CT, one with PET-CT, one with barium swallow and one with conventional radiography (CR). The CT findings at acute presentation showed the antrum lying above the diaphragm and the gastroesophageal junction (GEJ) and dilated fundus below the diaphragm in all seven cases (i.e., mesentero-axial according to the topographic classification of GV) (Figure 1D,E, Figure 2C,D, Figure 3B,C, Figure 4B; Table 1).

As shown in Table 2, all patients except one presented with both severe vomiting and epigastric/lower chest pain. In three of these patients (42.9%), inability to pass a nasogastric tube (NT) also occurred (Borchardt’s triad). Laboratory findings showed significantly elevated white blood cell count (>10,000 cells/μL) in five cases. One patient was found to have a significantly decreased WBC count (<4500 cells/μL), but this was judged to be due to a known diagnosis of lymphoma. Regarding complications, CT findings demonstrated stomach microperforation in three cases, always involving the fundus, with associated gastric wall pneumatosis in one of them. These findings were confirmed in subsequent emergency surgery. In fact, all patients were surgically managed, with complete or partial stomach resection being required in three patients. Five patients survived and remain asymptomatic to date (mean, 21.4 months; SD, 11.82; range, 7–36 months), one patient died during the postoperative period and another patient died 7 months later due to unrelated reasons.

## 4. Discussion

Despite the more than 300 cases of GV that have been published in the literature [12] and the significant improvement of imaging techniques in recent decades, the pathophysiology of GV has not been fully elucidated [17]. One of the main problems motivating this poor understanding of GV is terminological inconsistencies and overlapping entities that are frequently confused, even by imaging experts. In fact, careful reading of the available literature reveals several misconceptions related to (i) the role of paraesophageal hernia (PH) in the pathophysiology of GV; (ii) the concept of an upside-down stomach; and (iii) the imaging findings that allow the diagnosis of the type of GV described in this series (i.e., back-and-forth stomach). Therefore, it is worth discussing some of these points in the light of the imaging findings of our series.

### 4.1. Pathophysiology of Acute Gastric Volvulus

#### 4.1.1. Association between GV and PH

In our series, all cases of GV originated from HHs containing the gastric fundus or the entire stomach. This is contradictory to previous reports, which have consistently reported that most GV cases are associated with PHs [16,18,19,20]. Despite the presumed importance of this type of hernia in the pathophysiology of GV, there are very few insights on this aspect in recent literature. Paradoxically, excellent descriptions on the role of PH in GV can be found in old works by Culver [21] and by Gerson and Lewicki [22]. The former wrote an exhaustive description on the process that leads to GV through a case series of four patients with identical mechanisms. According to Culver, PH in GV develops through a diaphragmatic defect that is contiguous but not continuous with the esophageal hiatus. Herniation of the cardias into the chest through the said defect would result in the fundus and antrum eventually being located in the thoracic cavity. Re-herniation of the antrum into the abdomen through the diaphragmatic defect would then cause a space compromise, leading to GV after peristaltic movements originating from the herniated antrum. Although this theory reproduces a plausible mechanism based on the imaging findings of our series, no diaphragmatic defect was observed in any of our patients, neither during imaging examinations nor intraoperatively. Thus, Culver’s theory is not fully supported by the findings present in our series.

Twelve years after the study by Culver, Gerson and Lewicki [22] shed more light on this topic. They published a series including two cases through which they elucidated the more likely pathophysiologic mechanism involved in GV, namely subdiaphragmatic redescent of the fundus with fundic distension and crowding of the hernial orifice. To our knowledge, this is the only existing publication in which the pathophysiologic theory of GV is supported by consistent imaging examinations. However, it should be noted that all imaging exams used by these authors were based on barium swallow studies (i.e., not multiplanar imaging), and that they did not provide information on the type of HH before the acute presentation. Remarkably, these authors did not make the mechanism underlying GV contingent upon PH.

The question arises: why is PH so frequently associated with GV? PH is defined by stomach herniation with the gastroesophageal junction (GEJ) remaining in its normal anatomical position (i.e., the gastric fundus herniates into the mediastinum) [23]. Although not explicit in this definition, it is virtually assumed that the GEJ never moved from its normal location. However, in the case of a caudal re-herniation of a previously herniated stomach, the GEJ is pulled downward, mimicking a PH. This explains the common misinterpretation of previously published series and is supported by the imaging findings in our series, in which all cases of GV occurred in previous sliding (i.e., not paraesophageal) HHs.

Despite the illustrative and pedagogical nature of the studies by Culver and by Gerson and Lewicki, the underlying mechanisms of GV seem to have had no significant impact on the current understanding of GV. In fact, very few publications have discussed this theory and only in a marginal manner (e.g., [22]). Nevertheless, when analyzing images presented in most publications reporting GVs, it seems that this mechanism is fairly common. Finally, we would like to emphasize that, regardless of the type of HH associated with GV, the development of the latter mainly depends on the lack of sufficient abdominal anchorage of the stomach. This point has been largely discussed in previous works, which highlighted that the stomach is a highly mobile organ whose mobility increases when the supporting ligaments become loose or detached, initiating a cascade of events that may lead to GV [15,24].

#### 4.1.2. Terminological Inconsistencies: Upside-Down Stomach, Chronic GV, Organoaxial GV

On the other hand, we would like to emphasize another controversial issue which concerns the concept of ‘upside-down stomach’ (UDS). Some of the sliding HHs that were present in our series would correspond to UDSs according to the definitions given by some authors, since there was a complete stomach herniation in the mediastinum with an inverted position of the lesser and greater curvatures. However, in our opinion, the term UDS has been used in a particularly inconsistent manner in the literature, being frequently interchanged with other terms, including ‘chronic GV’, ‘organoaxial GV’, and even ‘paraesophageal hernia’. For instance, Umemura et al. [23] pointed out that the UDS is usually caused by organoaxial volvulus, referencing a study by Gryglewski et al. [25], in which they present a case of ‘incarcerated UDS’ that is quite reminiscent of the cases in our series. According to some authors, UDS is a type of mixed (i.e., type 3) HH, whilst for others it is a different type of hernia [26]. Other authors have described it as a ‘type of large paraesophageal hernia’ [27]. In the above-mentioned article by Umemura et al. it is stated that organoaxial volvulus ‘is often called UDS’. Similarly, al Daoud et al. [19] stated that ‘paraesophogeal hernias (…) show up as an inverted stomach’, which coincides with the description by Carter et al., who refer to PH directly as UDS [15]. To top it all off, some authors have discussed the concept of ‘chronic GV’ and have posed the debate on whether chronic intrathoracic VG is -an end-stage of the evolution of a sliding hernia or an extension of a true type II paraesophageal hernia’, which remains unresolved to date [28]. For these reasons, we classified all patients in our series as having sliding hiatal hernias, regardless of the degree of torsion in their thoracic cavity.

These are just but a few examples that illustrate the obvious terminological inconsistencies and heterogeneous definitions of these concepts. However, beyond the terminological debates, one might wonder: should the finding of an entire herniated stomach result in surgical correction to prevent the development of GV? In this regard, radiologists should be aware of current controversies regarding the management of large HHs. It is crucial to distinguish between symptomatic PH and asymptomatic or minimally symptomatic HH. According to Collet et al. [29], only patients with symptomatic hernias should undergo surgery, and prophylactic repair to prevent acute incarceration should only be undertaken in patients younger than 75 in good condition. In addition, authors such as Andolfi et al. [30] have suggested that asymptomatic patients younger than 50 should also be considered for surgery, provided that a comprehensive review of the risks, benefits and alternatives available is thoroughly discussed with the patients. In conclusion, current opinions seem to advocate offering elective surgery to all symptomatic patients and to asymptomatic individuals at low operative risk. Nevertheless, more studies are needed to address the unanswered questions regarding the optimal management of large PHs [31].

#### 4.1.3. Pathophysiology of GV: The ‘Back-and-Forth Stomach’

**Figure 5:**
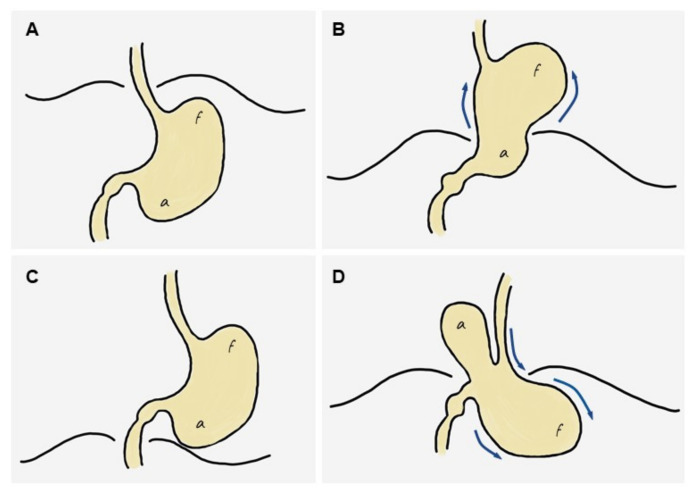
Diagram of the stages leading to the ‘back-and-forth’ stomach. (**A**) Normal stomach. (**B**) A portion of the cardias/fundus slides upwards into the mediastinum, leading to a sliding hiatal hernia, which increases progressively over time to eventually include most or all of the stomach in the mediastinum (including the antrum). (**C**) The entire stomach is located in the mediastinum and may rotate horizontally, predisposing the fundus to re-herniate into the abdomen. (**D**) A downward re-herniation of the fundus into the abdominal cavity through the esophageal hiatus occurs; the antrum normally lies above the diaphragm. The inability of the fundus content to be drained through the hernia neck leads to acute obstruction, i.e., gastric volvulus.

Based on the previous literature and on the imaging findings in our series, we propose a simplified mechanism explaining the development of GV from an HH. First, a sliding HH originates (Figure 5A), which progressively increases in size until it includes a large part or all of the stomach (including the antrum) in the thorax (Figure 5B,C). At this site it usually undergoes an axial rotation, predisposing the fundus to re-herniate into the abdomen, although horizontal rotation is not always present. Finally, there is a downward re-herniation of the fundus into the abdominal cavity through the esophageal hiatus, giving rise to obstruction due to inability to drain its content, and thus triggering acute GV (Figure 5D). The re-herniation of the fundus could be secondary to a sudden increase in intrathoracic pressure. It is logical to think that the three cases in our series in which an HH containing only the fundus was demonstrated probably evolved into a hernia containing the entire stomach since the antrum was located above the diaphragm in the acute presentation. In fact, the mean time from diagnosis of HH to the development of GV was similar in the group of patients in whom the hernia was demonstrated to contain only the fundus (x = 6.3 years, SD = 3.1) in comparison with those containing the entire stomach (x = 3.5 years, SD = 3.1). This supports the hypothesis that patients with HH containing the fundus eventually developed a complete HH prior to the development of GV, but this should be confirmed in future studies.

This pathogenesis gives rise to a highly specific CT semiology, characterized by the presence of the antrum lying above the diaphragm and the fundus below (usually dilated in the absence of NGT aspirate). We believe that these findings should be interpreted unequivocally as a sign of GV in the clinical context of acute epigastric or low chest pain.

### 4.2. Clinical Features, Laboratory Abnormalities and Outcomes of GV in Our Series

Regarding the presenting symptoms, these depend on the rapidity of onset, degree of gastric rotation, amount of obstruction and final position of the stomach [1,8,12,32,33,34]. Our series is in agreement with previous studies [9,35], with epigastric or lower thoracic abdominal pain associated with vomiting being the most frequent clinical presentation and common in all cases. Borchardt’s triad, which includes the inability to pass the NGT, was found in three cases (42.9%), a significantly lower percentage than that described in classic series (70%) [7]. This could suggest that this type of GV allows better passage of the NGT than other subtypes and is in agreement with previous studies [21].

Abnormalities in blood parameters described in the literature are very broad and varied, and they include elevated aldolase, CK [36], amylase, alkaline phosphatase (secondary to a kinking of the common bile duct when the duodenum is rotated) [37] and elevated white blood cell count [21]. The findings in our series show leukocytosis with neutrophilia and discrete increase in CRP values in all cases.

Regarding patient management, all cases in our series were treated surgically by laparotomy, gastropexy and/or partial resection of the stomach, with or without fundoplication. These procedures represent the most traditional management of the acute episode [30,34], although other techniques have been described (e.g., gastrojejunostomy, Opolzer’s procedure, Tanner’s procedure) [20]. Endoscopic decompression and gastrostomy, described in elderly [1] and high-surgical-risk [38] patients as a conservative method of management, was not performed in any of our patients. Of note, these conservative strategies are not without risk since there is a significant risk of perforation [7], and cases of GV have even been reported in patients with gastrostomy [9,39]. All the approaches were performed by laparotomy, although the use of laparoscopy has been described in the literature since 2004 [40] in high-risk elderly patients [37,38], and successful outcomes have been reported using endoscopic techniques [41]. Currently, some authors consider it a safe and acceptable approach [34]. Finally, mortality associated with acute GV is variable. Classic series report mortality rates of 30–50% [18], while more recent series estimates are around 15–20% [28]. In our series, only one of the patients died during the postoperative period, which makes up 14.2% mortality, in agreement with mortality estimates.

### 4.3. Limitations

This study has several limitations. First, it is retrospective in nature; thus, it is possible that some data regarding clinical presentation were not collected, and some previous imaging studies do not optimally show the type of HH. In addition, we lack imaging correlates of the entire stomach being herniated into the thorax prior to GV in three patients. However, imaging findings at acute presentation were analogous in all patients, and the time from diagnosis of HH to development of GV was longer in patients with fundus-only HH compared to entire-stomach HH. These observations support the hypothesis that the underlying mechanism of GV is shared by all patients in our study. Another limitation concerns the small sample size, which limits the generalizability of our conclusions despite the specificity of the findings. Finally, no histologic information of the surgical specimens was available, which would have allowed us to better correlate the ischemic findings seen on CT. The main strength compared to previous studies is the possibility of having previous imaging studies that allow us to demonstrate the proposed pathophysiological mechanism.

## 5. Conclusions

We have presented a series of cases of GV that confirms a theory that was proposed more than half a century ago but which has been largely ignored in subsequent publications. In addition, a number of overlapping entities and the inconsistent use of terminology has generated much confusion in the understanding of GV.

The pathophysiological mechanism that underlies these types of GV is the following: first, a sliding HH occurs, which progressively increases until it includes a large part or all of the stomach in the mediastinum. Then, the stomach rotates horizontally, predisposing the fundus to re-herniate to the abdomen. Finally, there is a caudal re-herniation of the fundus, triggering GV. This mechanism is reflected by a highly specific CT semiology, characterized by the presence of the antrum above the diaphragm and the fundus below. These findings should be interpreted unequivocally as a sign of GV in the clinical context of acute pain.

Given the terminological discrepancies in the literature, we believe it is appropriate to introduce and extend the term ‘back-and-forth stomach’ in view of imaging findings reflecting the above-explained mechanism. Further research is needed to determine the specificity of this presentation of GV in terms of symptoms, diagnosis, complications and management.

## Figures and Tables

**Table 1:** Sociodemographic data, hiatal hernia (HH) characteristics and type of acute gastric volvulus (GV). M, male; F, female; (PET-)CT: (positron emission tomography)-computed tomography; BS: barium swallow; CR: conventional radiography; WBC, white blood cell; GEJ, gastroesophageal junction.

Case	Sex	Age	History of HH	Type of HH	Part of the Stomach Herniated Prior to GV	Type of GV
1	M	76	10 years [symptomatic]	Sliding [barium swallow]	Fundus	Mesentero-axial (antrum above diaphragm and GEJ)
2	M	67	1 month [incidental]	Sliding [PET-CT]	Entire stomach	Mesentero-axial (antrum above diaphragm and GEJ)
3	F	69	5 years [symptomatic]	Sliding [CT]	Fundus	Mesentero-axial (antrum above diaphragm and GEJ)
4	M	81	7 years [incidental]	Sliding [CT]	Entire stomach	Mesentero-axial (antrum above diaphragm and GEJ)
5	M	69	2 years [symptomatic]	Sliding [CT]	Entire stomach	Mesentero-axial (antrum above diaphragm and GEJ)
6	F	85	5 years [symptomatic]	Sliding [CT]	Entire stomach	Mesentero-axial (antrum above diaphragm and GEJ)
7	F	47	4 years [incidental]	Sliding [CR]	At least fundus ^1^	Mesentero-axial (antrum above diaphragm and GEJ)

^1^ In this patient the HH was diagnosed by CR findings; thus, the exact amount of stomach herniated is unknown (see Figure 1).

**Table 2:** Clinical and laboratory parameters at acute presentation, complications and associated findings on imaging, treatment and outcomes of patients in our series. GV, gastric volvulus; LDH, lactate dehydrogenase; WBC, white blood cell; CRP, C-reactive protein; bpm, beats per minute; NG, nasogastric tube.

Case	Clinical Presentation (GV)	Blood Test Workup (GV)	Complications of GV	Relevant Associated Findings	Treatment	Outcome
1	Dark vomits, abdominal pain, food and fluids intolerance, dehydration, tachycardia [100 bpm]	WBC count [16,700/μL], CRP [12.0 mg/L], LDH [968 U/L]	Microperforation of fundus	-	Surgery [partial resection + fundoplication]	Died during post-operative period
2	Abdominal and lower chest pain, nausea and vomiting, fever [38.5 °C], inability to pass NG tube (Borchardt’s triad)	WBC count [660/μL], CRP [41.4 mg/L], LDH [542 U/L]	-	Lymphadenopathies (lymphoma)	Surgery [partial resection + fundoplication]	Alive (died 7 years later due to lymphoma)
3	Abdominal and lower chest pain, severe vomiting	WBC count [12,370/μL], CRP [46.9 mg/L], K+ [3 mEq/L]	-	Prostatic tumor	Surgery [fundoplication + cardioplasty]	Alive (4 years follow-up)
4	Intense abdominal pain, severe vomiting, tachycardia [150 bpm], signs of peritonitis, inability to pass NG tube (Borchardt’s triad)	WBC count [18,730/μL], CRP [2.7 mg/dL] Lactic acid [69.6 mg/dL] K+ [2.9 mEq/L]	Gastric pneumatosis and microperforaation of fundus	Splenic laceration, left hernioplasty	Surgery [partial resection + fundoplication + cardioplasty + splenectomy]	Alive (2 years follow-up)
5	Abdominal pain, severe vomiting, inability to pass nasogastric tube (Borchardt’s triad)	WBC count [14,980/μL], CRP [13 mg/L]	-	-	Surgery [cardioplasty + fundoplication + jejunostomy]	Alive (3 years follow-up)
6	Dark vomits, food and fluid intolerance, tachycardia [100 bpm]	WBC count [9,070/μL], LDH [286 U/L]	-	-	Surgery [cardioplasty + fundoplication]	Alive (2 years follow-up)
7	Abdominal pain, vomitting, mass in left hypochondrium	WBC count [21,670/μL] CRP [35 mg/L] K+ [2.2 mEq/L] Cl− [76 mEq/L]	Microperforation of fundus	-	Surgery [Hernia repair + fundoplication]	Alive (3 years follow-up)

## Data Availability

All data related to the case series presented in this article are available from the corresponding author upon reasonable request.
